# Effects of Carbamazepine and Phenytoin on Pharmacokinetics and Pharmacodynamics of Rivaroxaban

**DOI:** 10.3390/pharmaceutics12111040

**Published:** 2020-10-30

**Authors:** Lien Thi Ngo, Sung-yoon Yang, Quyen Thi Tran, Sang Kyum Kim, Hwi-yeol Yun, Jung-woo Chae

**Affiliations:** College of Pharmacy, Chungnam National University, Daejeon 305764, Korea; lienngovn@cnu.ac.kr (L.T.N.); 201851000@o.cnu.ac.kr (S.-y.Y.); quyentran@cnu.ac.kr (Q.T.T.)

**Keywords:** rivaroxaban, carbamazepine, phenytoin, drug interaction, pharmacokinetics, pharmacodynamics

## Abstract

Rivaroxaban (RIV) is commonly prescribed with carbamazepine or phenytoin (CBZ/PHT) in post-stroke seizure or post-stroke epilepsy patients. Although adverse events have been reported in several previous studies when they are coadministered, there are no studies of the interactions between these drugs. Therefore, our study was conducted to solve this lack of information. The potential effects of CBZ/PHT were investigated by comparing the pharmacokinetic (PK) and pharmacodynamic (PD) parameters of RIV between the control group (RIV alone) and the test groups (RIV administered with CBZ/PHT) in rats using the noncompartmental analysis (NCA) and the compartmental model approach. The NCA results indicate that *AUC_t_* of RIV decreased by 57.9% or 89.7% and *C_max_* of RIV decreased by 43.3% or 70.0% after administration of CBZ/PHT, respectively. In addition, both CBZ and PHT generally reduced the effects of RIV on the prothrombin times of the blood samples. PK profiles of RIV were most properly described by a two-compartment disposition model with a mixed first- and zero-order absorption kinetics and a first-order elimination kinetics. The compartmental model approach showed that a 211% or 1030% increase in *CL/F* of RIV and a 33.9% or 43.4% increase in *D*2 of RIV were observed in the test groups by the effects of CBZ/PHT, respectively. In conclusion, CBZ and PHT significantly reduced RIV exposure and therefore reduced the therapeutic effects of RIV. Consequently, this might result in adverse events due to insufficient RIV concentration to attain its therapeutic effects. Further studies are needed to validate this finding.

## 1. Introduction

Ischemic stroke is a medical emergency, and without timely intervention, it can lead to severe complications. Several different diseases can cause ischemic stroke. Atrial fibrillation is one of the common causes and accounts for up to 20% of ischemic strokes annually [1]. To reduce the risk of stroke after atrial fibrillation, anticoagulants, including vitamin K antagonists (VKA) and direct oral anticoagulants (DOACs), are usually recommended. VKA and DOACs prevent the formation of blood clots and subsequently reduce the chances of developing strokes. Recently, DOACs have been preferred to VKA for the treatment of stroke and systemic embolism in patients with nonvalvular atrial fibrillation due to their safety and advantages [2]. Various studies reported that treatment with DOACs is associated with a reduction in mortality (e.g., cardiovascular mortality, myocardial infarction, ischemic stroke) [3,4], major bleeding [5,6,7,8], stroke [4,9], and no requirement of laboratory monitoring. DOACs are divided into two groups according to mechanism: direct thrombin inhibitors (e.g., dabigatran) and direct Xa inhibitors (e.g., apixaban, edoxaban, and rivaroxaban (RIV)) [10].

Stroke is one of the most common causes of seizures [11]. The incidence of a seizure occurring in early stroke stage (within two weeks of stroke) is 2.2–33% and in late stroke stage (at least two weeks after stroke) is 3.0–67% [12]. Seizure after stroke is usually controlled by anti-epileptic drugs (AEDs); therefore, in patients with stroke-related seizures, a combination of DOACs and AEDs is required. AEDs are classified into first-, second- and third-generation AEDs [13]. First-generation AEDs have been recommended as the first line for treatment of seizures in patients with stroke due to their efficacy and low cost [14,15]. They also have been reported as strong inducers of cytochrome (CYP) 450 [16] and P-glycoprotein (P-gp) transporters [17,18,19,20,21,22], so consequently, they decrease exposures of coadministration drugs that are substrates of CYP450 and/or P-gp [23,24].

As DOACs have been reported to be substrates of CYP450 and/or P-gp transporters [25,26], drug–drug interaction (DDI) possibilities could be expected when they are coadministered. Several adverse events that occur when DOACs are concomitantly administered with first-generation AEDs have been previously reported [27,28,29,30]. Stöllberger and Finsterer [29] reported that venous thrombosis recurred in a 55-year-old male who was treated with RIV 20 mg/day for 4 months (for unprovoked venous thrombosis) and carbamazepine (CBZ) 900 mg/day for years (for epilepsy with complex partial seizures and secondary generalization). The venous thrombosis was controlled after 5 days when therapy with RIV was stopped and switched to low-molecular-weight heparin followed by phenprocoumon. Becerra et al. [30] reported the first case of laboratory interaction between RIV and phenytoin (PHT) in a patient with cerebral venous thrombosis. After one week of treatment with RIV (30 mg/day) and PHT (300 mg/day), the anti-Xa levels in the patient were considerably lower, such that they did not achieve the reference value (100 ng/mL–300 ng/mL). Due to concerns about thrombosis progression, RIV was switched to dabigatran. After that, the status of asymptomatic condition and thrombin time (PT) (>180 s) was controlled.

To the best of our knowledge, there were no studies conducted to investigate the effects of CBZ or PHT (CBZ/PHT) on both the pharmacokinetics (PK) and the pharmacodynamics (PD) of RIV. Only one study was reported the effects of PHT on the PK of RIV; however, no information regarding administered doses was reported [31]. Our study was, therefore, conducted to solve the lack of information. Potential effects of CBZ and PHT on PK and PD of RIV were determined by comparing PK and PD parameters of RIV in the control group (RIV given alone) and test groups (RIV given with CBZ/PHT) using both the noncompartmental analysis (NCA) and the compartmental model approach.

## 2. Materials and Method

### 2.1. Materials

Rivaroxaban (purity >99%) was purchased from ChemScene (Monmouth Junction, NJ, USA). CBZ, carboxymethylcellulose, domperidone, and PHT sodium were purchased from Sigma-Aldrich (St. Louis, MO, USA). Formic acid was acquired from the SAMCHUN pure chemical company (Pyeongtaek, Republic of Korea). High performance liquid chromatography-grade methanol was obtained from Thermo Fisher Scientific (Waltham, MA, USA). Water was obtained using an option-Q purification system (Elga Ltd., High Wycombe Bucks, UK). All other reagents were analytical grade.

### 2.2. Study Design

Our study was conducted in 24 male Wistar rats, which were purchased from Orientbio (Seongnam, Republic of Korea). The experiments were planned under the approval of the Animal Ethics Committee of Chungnam National University (NO. 2019012A-CNU-193, (approved on 27 December 2019). All procedures were conducted in accordance with the assurance statement and guidelines in the National Institutes of Health’s *Guide for the Care and Use of Laboratory Animals*.

Rats were weighed and randomly divided into three groups: GR1—control group (*n* = 12), RIV administered with saline; GR2—CBZ treated group (*n* = 6), RIV administered with CBZ; and GR3—PHT treated group (*n* = 6), RIV administered with PHT. Before administration, RIV, CBZ, and PHT were dissolved in corn oil, 0.6% carboxymethylcellulose solution, and water, respectively, to make solutions with final concentrations of 5, 3, and 5 mg/mL, respectively. Starting the first day, each rat was pretreated twice daily with saline (GR1), CBZ 45 mg/kg (GR2), or PHT 15 mg/kg (GR3) for six consecutive days. On Day 7, RIV 3 mg/kg was given 30 min after the administration of saline/CBZ/PHT.

For the PK analysis, blood samples (200 μL) were collected in heparinized tubes before treatment and then subsequently at 0.25, 0.5, 1, 2, 4, 8, 10, and 24 h after treatment of RIV on Day 7. Plasma was separated after centrifugation (15,000 rpm, 10 min) and stored at −80 °C until analysis. For the PD analysis, a 750 μL blood sample was taken in 3.2% sodium citrate-contained tubes before treatment and then subsequently at 1, 4, 8, and 10 h after treatment of RIV on Day 7. Samples were centrifuged at 5500 rpm for 10 min. The supernatant was collected and stored at −4 °C until analysis. The PT was measured within 24 h of the collection.

### 2.3. Plasma Concentration and PT Measurement Methods

Plasma concentration of RIV, CBZ, and PHT was determined by validated methods using high-performance liquid chromatography (1200 series HPLC; Agilent, Santa Clara, CA, USA) coupled with tandem mass spectrometry (Qtrap 4000; Sciex, Framingham, MA, USA) [32,33,34]. Chromatographic separation of analytes was conducted using a GEMINI-NX C18 column (50 mm × 2 mm; 5 μm; Phenomenex, Torrance, CA, USA) with a gradient elution of mobile phase, which contains 0.1% formic acid solution (A) and methanol (B) at a flow rate of 0.3 mL/min. The analysis was performed with an electrospray ionization probe in the positive ion mode. The ion spray voltage was 5500 V, and the source temperature was 450 °C. Multiple reaction monitoring transitions of each analyte were as follows: m/z 436.2 → 144.9 for RIV, m/z 238.3 → 194.0 for CBZ, m/z 253.2 → 182.1 for PHT, and m/z 428.4 → 175.1 for domperidone (internal standard). Calibration curves were linear in the range of 10–1000 ng/mL, 100–25,000 ng/mL, and 100–25,000 ng/mL for RIV, CBZ, and PHT, respectively (R^2^ > 0.99).

PT was measured using a blood coagulation analyzer (ACL 7000, Instrumentation Laboratory, Bedford, MA, USA) at Shinwon Medical Foundation (Daejeon, Korea) using PT recombiPlasTin 2G reagent (Instrumentation Laboratory, Bedford, MA, USA).

### 2.4. Noncompartmental PK and PD Analysis

Before performing NCA, the below limit of quantification (BLQ) data were handled [35]. If a BLQ value in the profile occurs after dosing time and before the first measurable concentration, it is set to zero. If a BLQ value occurs after the last quantifiable concentration or between two measurable concentrations, it is treated as missing data. If two BLQ values occur in succession after maximum plasma concentration (*C*_max_), subsequent concentrations are omitted.

The PK and PD parameters were calculated using Phoenix WinNonlin (version 8.2.0.4383, Certara L.P, Princeton, NJ, USA). For the PK parameters, *C*_max_ and time to reach *C*_max_ (*T*_max_) were obtained directly from observations. Area under the curve (*AUC*) from zero to the last measurement (*AUC*_t_) was calculated using a linear log trapezoidal method. *AUC* from zero to infinity (*AUC*_inf_) was the sum of *AUC*_t_ and extrapolated *AUC* from the last time point to infinity, *AUC*_inf_ = *AUC*_t_ + *C*_last_/*k_el_*, where *C*_last_ is the last plasma concentration and *k_el_* is the terminal rate constant deriving from the slope of linear regression log-transformed of plasma concentration. The apparent clearance (*CL/F*) was derived as dividing the administered dose by *AUC*_inf_, *CL/F* = dose/*AUC*_inf_, and the apparent volume of distribution (*V/F*) was calculated as dividing *CL/F* by the terminal rate constant (*V/F* = *CL/F/k_el_*), where *F* is bioavailability. For the PD parameters, the maximum percentage increase in PT (*E*_max_) and area under the time course of PT (*AUC*_PT_) were reported. *AUC*_PT_ was calculated using a linear log trapezoidal method. *E*_max_ was calculated from the maximum PT value (*PT*_max_) and PT value at a time of zero (*PT*_zero_), as described in the following equation.

*E*_max_ = (*PT*_max_ − *PT*_zero_)/*PT*_zero_ × 100% (1)

The comparison of RIV parameters between (GR1 with GR2) and (GR1 with GR3) was tested with the Mann–Whitney U test, performed on the SPSS software version 24.0 (IBM SPSS Statistics, IBM Corporation, Armonk, NY, USA). The statistically significant level was set at *p* < 0.05.

### 2.5. Population PK Analysis

#### 2.5.1. Development of a Base Model

PK data of RIV were analyzed using NONMEM version 7.3.0 executed through the Perl-speaks-NONMEM (PsN) toolkit (version 5.0.0, 2020) integrated into Pirana software (version 2.9.7, Certara L.P, Princeton, NJ, USA, 2017) [36]. The first-order conditional estimation (FOCE) method with the interaction option method was applied to each step of the model development for RIV [37]. The first-order kinetics was assumed to describe the elimination. Random error (inter-individual variability) was tested for each parameter. Residual random error (intra-individual variability) was described by a combined proportional and additive error model. First- or zero-order kinetics was applied to describe the absorption of RIV from the GI tract into the systemic circulation. Then, with the assumption that the absorption of RIV occurs via two pathways, a combination of a first- and zero-order absorption model was developed. In detail, a fraction of RIV dissolved in the GI tract fluid was absorbed from the GI tract into the systemic circulation via the hepatic portal vein. This process was modeled by zero-order kinetics with a duration absorption time of *D*2. The remaining RIV accompanied by corn oil was absorbed directly into the systemic circulation by collecting through the intestinal lymphatic system. This process was modeled by first-order kinetics with an absorption constant *Ka*.

**Figure 1:**
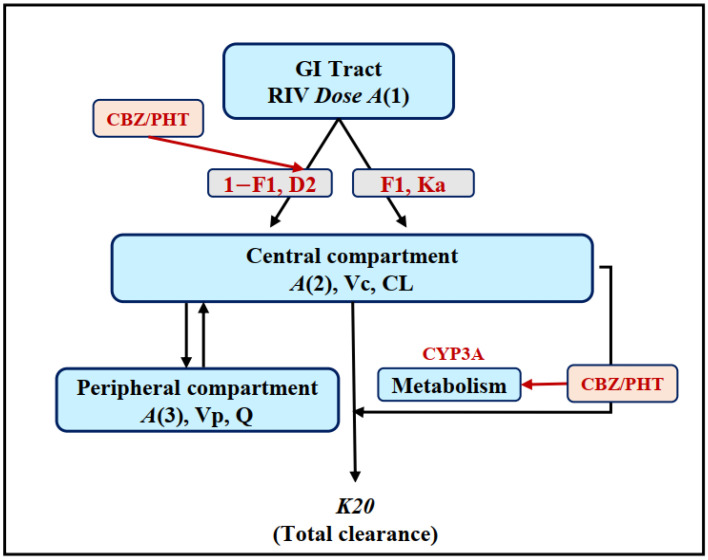
Schematic representation of the base population pharmacokinetic model for rivaroxaban (RIV) and strategies to investigate the effect of carbamazepine or phenytoin (CBZ/PHT) on the pharmokinetics (PK) of RIV. All notations are mentioned in the text.

Only PK data of RIV administered in the control group (GR1) were used for the development of the base model. The effects of CBZ/PHT on the PK of RIV were investigated based on the full dataset of RIV (GR1, GR2, and GR3). The proposed base PK model for RIV is illustrated in Figure 1.

#### 2.5.2. Development of a Covariate Model with Investigation of CBZ/PHT Effects

Effects of CBZ/PHT on PK of RIV were tested by introducing the pretreatment of CBZ/PHT as a covariate on the apparent clearance (*CL/F*) and duration absorption time (*D*2). The covariate was coded in the model as an index variable with the number of 1, 2, and 3 standing for GR1, GR2, and GR3, respectively. For example, the categorical covariate was added into the model to determine the effect of CBZ/PHT on the apparent clearance of RIV, as described in the equation below.

*CL* = *TVCL* × (1 + *FCL*_group_) (2)

In the above equation, *TVCL* is the typical value of the clearance of RIV in subjects belonging to GR1 and *FCL*_group_ is the fractional change in the clearance of RIV, which receives different values according to the subgroup of subjects. Covariate model building was accomplished by mixed stepwise forward addition (*p* < 0.01) and stepwise backward elimination (*p* < 0.05), based on the change in the objective function value (OFV) as well as reductions in the interindividual variability (IIV) of PK parameters. The log-likelihood ratio test was used to discriminate the two nested models. A decrease of 3.84 units (one degree of freedom) or 5.99 units (two degrees of freedom) in the OFV was considered as a significant difference at the level of *p* < 0.05. A decrease of 6.62 units (one degree of freedom) or 9.20 units (two degrees of freedom) in the OFV was considered as a significant difference at the level of *p* < 0.01.

#### 2.5.3. Selection and Evaluation for the Final Model

The final model for RIV was selected based on the degree of OFV, the precision of estimated parameters, and goodness of fit plots. The selected model was then evaluated by both visual predictive check (VPC) and nonparametric bootstrap analysis. In detail, for the VPC, 1000 concentration–time datasets of RIV were simulated based on the developed population PK model. Study design, parameters, their relative standard deviations (RSD), and random effects of RIV in the simulated populations were assumed to be the same as those from the observed population [36]. After the simulation, the 95% confidence interval (CI) of the median and 5th and 95th percentiles of the simulated concentrations were plotted accompanied by the observed data for VPC. For the bootstrap analysis, 1000 concentration–time datasets of RIV were generated by randomly selected samples from the observed dataset with replacement. After generating these datasets, the developed PK model was fitted to each dataset. The median and 90% CI of all model parameters from 1000 simulated datasets were calculated and compared with those from the observed dataset to assess the parameter uncertainty [36].

## 3. Results

### 3.1. Noncompartmental PK and PD Analysis

**Figure 2:**
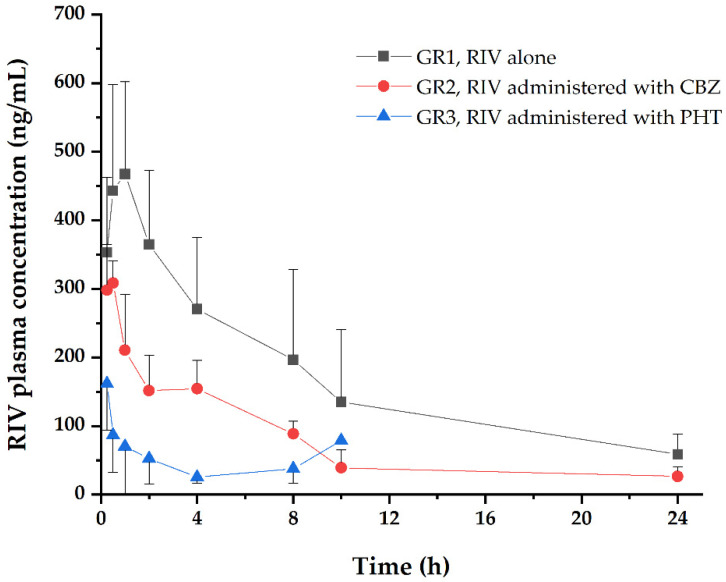
Plasma concentration–time profile of RIV after oral administration of RIV with/without CBZ/PHT pretreatment. Each point represents the mean and standard deviation.

Figure 2 shows the profile of plasma concentration versus time of RIV with/without pretreatment with CBZ/PHT. The corresponding RIV PK parameters are listed in Table 1.

All PK parameters of RIV showed significant (*p* < 0.01) changes when RIV was pretreated with CBZ or PHT compared with RIV administered alone. *AUC*_t_ of RIV significantly (*p* < 0.01) decreased from 3088 to 1299 ng/mL×h by the effects of CBZ and from 3088 to 317 ng/mL×h by the effects of PHT. In addition, *C*_max_ and *T*_max_ of RIV also significantly (*p* < 0.01) decreased.

**Figure 3:**
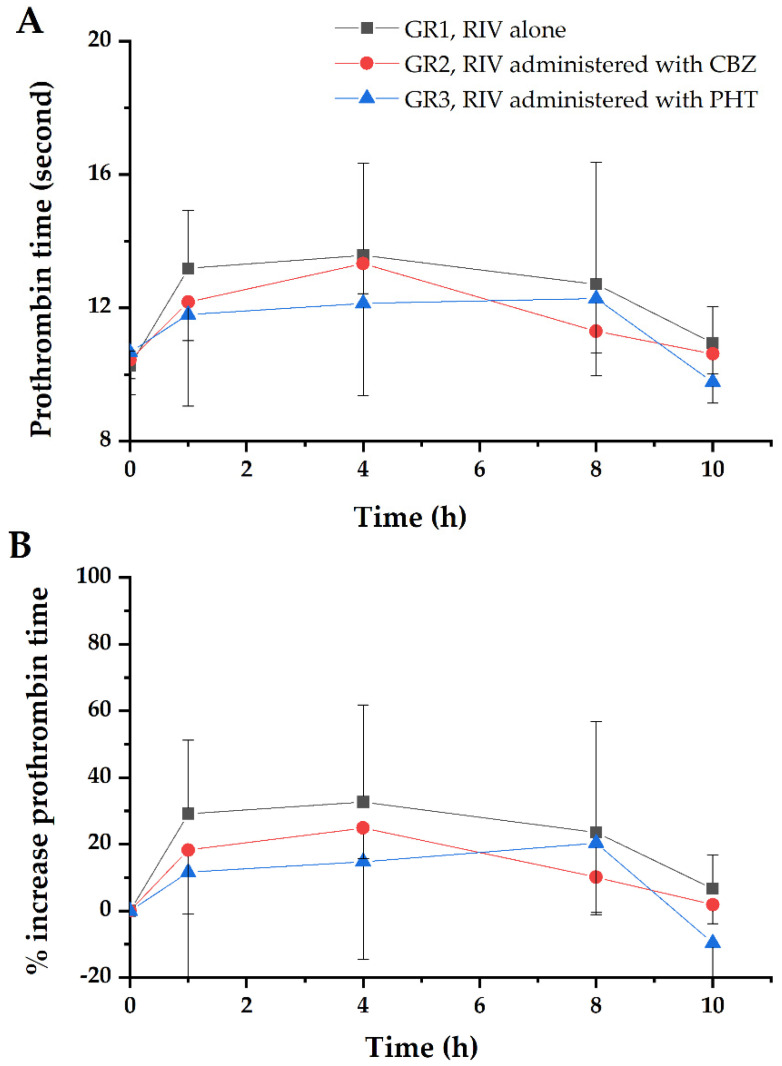
(**A**) PT profile and (**B**) percentage increase in PT of the blood samples under the control of RIV with/without CBZ/PHT pretreatment. Each point represents the mean and standard deviation.

The time course of PT and percentage increase in PT are illustrated in Figure 3. The corresponding PD parameters are listed in Table 2. The effects of CBZ/PHT caused a declining trend in the PT of the blood samples under the control of RIV. *AUC*_PT_ decreased 25.4% (from 212%×h to 158%×h) and 37.9% (from 212%×h to 132%×h) with CBZ and PHT pretreatment, respectively. However, these decreases were not statistically significant (*p* > 0.05).

### 3.2. Population PK Analysis

#### 3.2.1. Development of the Population Pharmacokinetic Model

Base model building steps for PK of RIV are summarized in Table 3. The PK of RIV in GR1 was used to develop the base model. The first- and zero-order kinetics used to describe the absorption of RIV were performed in both one- and two-compartment disposition models. IIV was tested for each PK parameter; however, only the addition of IIV for *CL/F* and $V_{c}$/*F* significantly decreased the OFV. As seen in Table 3, a two-compartment model with combined first- and zero-order kinetics used to describe the absorption was selected as the final base model. This model was then applied to describe the full PK dataset of RIV. Estimated parameters, their relative standard errors (RSE), and residual errors are listed in Table S1 (Supplementary Materials).

#### 3.2.2. Development of a Covariate Model with Investigation of CBZ/PHT Effects

This final base model was further developed and applied to investigate the effects of CBZ/PHT on PK of RIV by testing the CBZ/PHT pretreatment as a covariate on RIV PK parameters. A decrease of 30.80 units in OFV and 10.1% in IIV of *D*2 were observed when the covariate effect on *D*2 was considered. In addition, a drop of 41.05 units in OFV and 79.0% in IIV of *CL/F* was observed when the covariate effects on *CL/F* were introduced. The final model was built with the introduction of the covariate effects on both *D*2 and *CL/F* (model no. 2.4). In summary, the effect of CBZ/PHT was investigated through covariate effect modeling as described in the equations below.

*CL/F* = 0.610 × (1 + *FCL*_group_) (3)

*D*2= 6.62 × (1 + *FD*2_group_) (4)

In the above equations, *FCL*_group_ is 0 for GR1, 2.11 for GR2, and 10.3 for GR3, and *FD*2_group_ is 0 for GR1, 0.339 for GR2, and 0.434 for GR3. An increase of 211% in *CL/F* and 33.9% in *D*2 was observed for RIV by the CBZ pretreatment, and an increase of 1030% in *CL/F* and 43.4% in *D*2 for RIV was observed for RIV by the PHT pretreatment. All estimated parameters, their RSE, and random errors are listed in Table 4.

#### 3.2.3. Evaluation of Population PK Model for RIV

**Figure 4:**
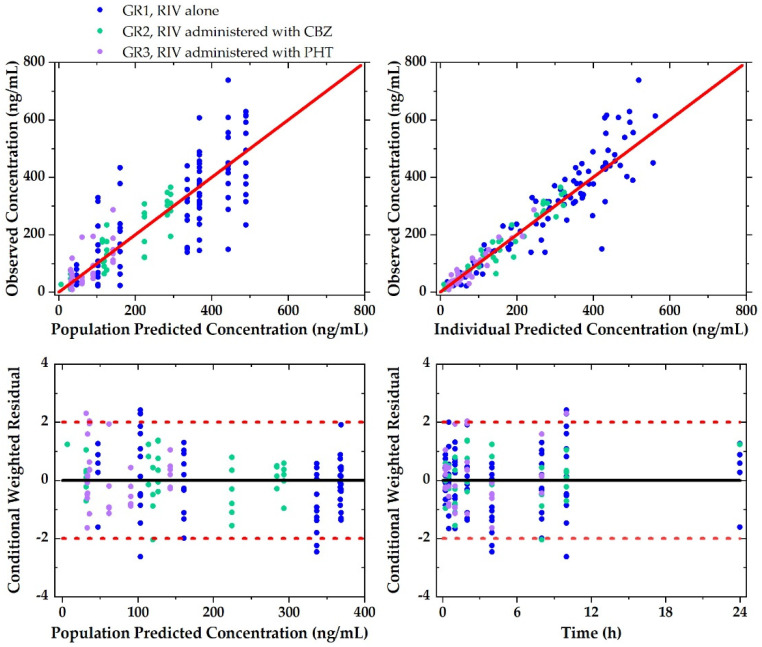
Goodness-of-fit plots of the final model on PKs of RIV.

**Figure 5:**
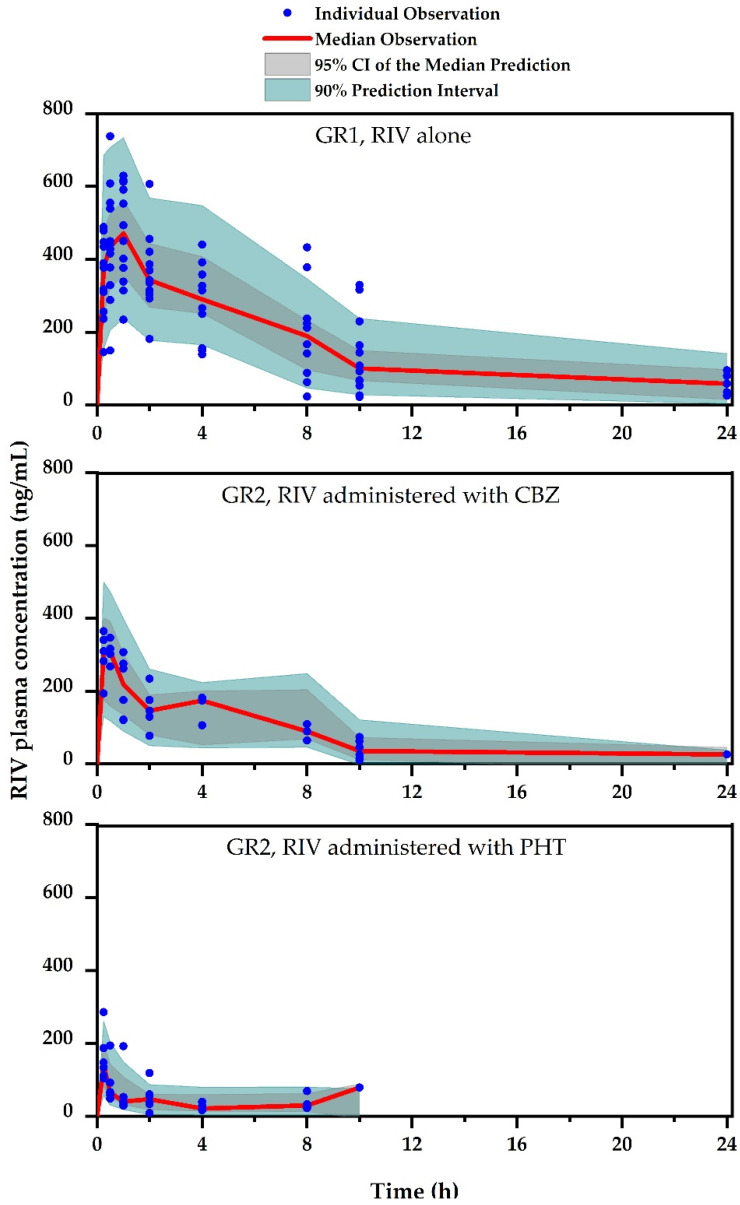
Visual predictive check for the final model on population PKs of RIV.

The basic GOF plots of the developed model are shown in Figure 4, suggesting that the model predicted individual observed concentrations well. VPC for the developed model (Figure 5) showed that a 90% prediction interval of simulated population covered the observed population concentrations of RIV well. In addition, 95% CI of simulated concentrations at the 5th, 50th, and 95th percentiles also covered the respective percentiles of the observed concentrations well. Additionally, the results of the bootstrap analysis indicate that parameter estimates obtained from the bootstrap replicates were all consistent with those obtained from the observed population, suggesting that the estimates were unbiased. In conclusion, the developed population model could be applied to describe the PK of RIV.

## 4. Discussion

Drug combination therapy is widely used in clinical practice, especially in elderly patients or patients with multiple comorbidities. In addition to the benefits, drug combinations may pose risks of drug interactions. Coadministration of RIV and CBZ or PHT is commonly prescribed to post-stroke seizure or post-stroke epilepsy patients. Drug interactions have been reported in some case studies with decreased RIV exposures, resulting in adverse effects, including an increased risk of bleeding or pulmonary embolisms [28,29,30,38,39]. However, there have been no studies performed in animals or humans to investigate potential effects of CBZ/PHT on both PK and PD of RIV. The present study was, therefore, conducted to solve the lack of information.

The study was conducted in three groups of rats, GR1 (RIV administered alone), GR2 (RIV pretreated with CBZ), and GR3 (RIV pretreated with PHT). The effects of CBZ/PHT on PK and PD of RIV was investigated by comparing the PK and PD parameters of RIV between the control and test groups. The NCA and compartmental model analyses were performed. The doses administered to the rats were selected based on the recommended doses for adults in clinical practice. According to the U.S. Food and Drug Administration, the recommended dosage of RIV for a reduction in risk of stroke in nonvalvular atrial fibrillation is 20 mg once daily [40]. For CBZ, the recommended maintenance dosage is 800–1200 mg daily for the indication of epilepsy [41]. For PHT, the recommended starting dosage is 300 mg daily, adjusting the dosage to suit individual requirements [42,43]. In summary, a human therapeutic dose for RIV, CBZ, and PHT of 20, 900, and 300 mg/day, respectively, corresponding to 0.333, 15, and 5 mg/kg/day (for an assumed human body weight of 60 kg) were applied to establish the doses in rats using a conversion factor based on the body surface area of humans and rats [44]. Accordingly, the doses in rats were determined at approximately 2, 90, and 30 mg/kg/day for RIV, CBZ, and PHT, respectively. Considering the effects of CBZ/PHT on RIV PK and the sensitivity of the analysis system, we increased the dose of RIV to 3 mg/kg.

The results of the NCA indicate that CBZ and PHT significantly (*p* < 0.01) affected PK of RIV. In detail, *AUC*_t_ of RIV decreased by 57.9% or 89.7% and *C*_max_ of RIV decreased by 43.3% or 70.0% due to the effects of CBZ/PHT, respectively. In addition, both CBZ and PHT generally reduced effects of RIV on PT of the blood samples, and consequently decreased PT was observed although the decreases were not significant (*p* > 0.05).

In addition to the NCA, a population compartmental model was also applied due to its advantages for assessing potential DDI [45], particularly for RIV, because RIV’s PK profile presents a double peak and/or multiple phases in its plasma concentration–time curve. In this case, NCA requires a large enough number of samples per subject to accurately determine PK parameters. Atypical PK profiles have been reported previously, but no conclusion about the phenomenon has been made [46,47]. In the present study, a population PK model was first developed, then, to investigate the effects of CBZ/PHT on PK of RIV, the CBZ/PHT pretreatment as a covariate was tested on RIV PK parameters. In general, first- or zero-order absorption kinetics is used to describe the absorption of a drug following oral administration. These conventional kinetics were performed on both one- and two-compartment models at an earlier step. However, they could not describe the observed PK of RIV well, so a model with multiple fractional phases was then developed. The assumption was that different absorption rates might lead to multiple peaks or phases in its PK profile. That was supported by population models developed previously for drugs showing the same phenomenon [48,49,50,51]. Given that solubility is a key factor governing the rate and extent of drug absorption, RIV is sparingly soluble in aqueous buffers. Its solubility is approximately 10 mg/L in both artificial stomach (pH 1–2) and artificial intestinal (pH 6.8) media [52]; in contrast, it is dissolved in various organic solvents [53]. Due to its poor solubility in both the stomach and intestine, zero-order kinetics (1-*F1*, *D*2) were developed to describe the absorption where RIV was moved from the GI tract, crossing the basolateral membrane, and entering the systemic circulation via the capillary network surrounding the enterocytes and hepatic portal vein. The absorption was limited by the amount of RIV solubilized in the GI fluids. In addition, as RIV was dissolved in corn oil for the administration, first-order kinetics (*F1*, *Ka*) were developed for the RIV absorption (accompanied corn oil) following the lipid absorption pathway, by which RIV entered the systemic circulation by being transported via the lymph through mesenteric lymph nodes and collecting lymphatic vessels [54]. This model described the PK profiles of RIV well. It was then applied to investigate the effects of CBZ/PHT on the PK of RIV.

RIV is metabolized via CYP450 3A4/3A5, 2J2, and CYP-independent mechanism. Among these, the contribution of CYP3A4/3A5 accounts for approximately 18% and CYP2J2 for approximately 14% of the total RIV elimination. In addition to the metabolism, the transporter-mediated renal excretion accounts for approximately 36% of the total renal excretion of the unchanged RIV drug. Meanwhile, both CBZ and PHT have been reported to be strong inducers of CYP450 and P-gp, which has been implicated in many drug interactions [17,18,19,20,21,22]. Therefore, when RIV and CBZ/PHT are coadministered, an increase in the RIV elimination (due to the induction of CYP450 and P-gp renal efflux transporters) and a decrease in the RIV absorption (due to the induction of P-gp intestinal efflux transporters) are expected. Therefore, the pretreatment of CBZ/PHT was tested as a covariate on the *CL/F* and *D*2 of RIV. The results show that CBZ exerted an increase of 211% in *CL/F* and 33.9% in *D*2 of RIV; similarly, PHT exerted an increase of 1030% in *CL/F* and 43.4% in *D*2 of RIV, compared with those in the control group. In comparison to the NCA results, the increase in *CL/F* by the presence of CBZ was closely consistent. In contrast, there was a difference in the effects of PHT. That might be explained by the fact that the RIV levels in the plasma of rats pretreated with PHT declined so fast that the observations were not enough to describe the elimination phase well.

Some limitations remained in the present study. First, the study was conducted on a small number of rats. In the case of the PD study, the coefficient of variations for each parameter were relatively large. For instance, they were 56.3%, 75.4%, and 96.6% for the *E*_max_ value of PT; therefore, a larger number of subjects is required to estimate these PD parameters accurately for a population. Second, a drug interaction study concerning the CYP3A4 metabolism using rat models has limitations due to interspecies difference of enzyme expression. The CYP3A subfamily is the most important of all human drug-metabolizing enzymes and is involved in the bio-metabolism of approximately 50% of marketed therapeutic drugs [41], although its content in the liver is only 30% of total CYP450. Among them, CYP3A4/3A5 are the most abundant CYP isoforms in the human liver [55]. Meanwhile, CYP3A4/3A5 is not one of the major isoforms in the rat liver, but rather CYP3A1 [56]. However, the results obtained in the present study do agree with those reported in case reports previously; therefore, the present study might predict the interactions between RIV and CBZ/PHT in humans. Further studies are needed to validate our study.

## 5. Conclusions

CBZ/PHT significantly decreased exposures of RIV when they were pretreated in rats. A 57.9% decrease (from 3088 to 1299 ng/mL×h) in *AUC*_t_ and a 43.3% decrease (from 540 to 306 ng/mL) in *C*_max_ were observed by the CBZ effects. A 89.7% decrease (from 3088 to 317 ng/mL*h) in *AUC*_t_ and a 70.0% decrease (from 540 to 162 ng/mL) in *C*_max_ were observed by the PHT effects. These decreases might be explained by the effects of CBZ/PHT on the RIV clearance and absorption through the induction in metabolism (CYP450) and P-gp efflux transporter. CBZ produced 211% and 33.9% increases in *CL/F* and *D*2 of RIV, respectively. PHT produced 1030% and 43.4% increases in *CL/F* and *D*2 of RIV, respectively. In addition to effects on PK, CBZ/PHT affects the PD of RIV, as indicated by decreases in PT of the blood samples; however, the decreases were not significant. Further studies are necessary for the validation of our findings.

## Figures and Tables

**Table 1:** PK parameters (mean ± standard deviation) of RIV with/without PHT/CBZ pretreatment and result of statistical analysis. GR1, RIV alone (*n* = 12); GR2, RIV administered with CBZ (*n* = 6); GR3, RIV administered with PHT (*n* = 6).

PK Parameters	GR1	GR2	GR3
^‡^*T*_max_ (h)	1.00 (0.250–8.00)	0.250 (0.250–0.500) *	0.250 (0.250–0.250) *
*C*_max_ (ng/mL)	540 ± 106	306 ± 62.4 *	162 ± 67.8 *
*AUC*_t_ (ng/mL*h)	3088 ± 1103	1299 ± 417 *	317 ± 188 *
*AUC*_inf_ (ng*h/mL)	3565 ± 1648	1595 ± 645 *	524 ± 234*
*V/F* (L/kg)	7.21 ± 3.58	14.9 ± 5.93 *	49.7 ± 27.6 *
*CL/F* (L/h/kg)	1.03 ± 0.495	2.19 ± 0.933 *	7.46 ± 5.27 *

Statistical analysis was performed to test the difference in PK parameters of RIV between GR1 with GR2 and GR1 with GR3. * *p* < 0.01; ^‡^*T*_max_ was presented as median (range).

**Table 2:** PD parameters (mean ± SD) of RIV with/without CBZ/PHT pretreatment. GR1, RIV alone (*n* = 11); GR2, RIV administered with CBZ (*n* = 6); GR3, RIV administered with PHT (*n* = 5).

Parameter	GR1	GR2	GR3
*E*max (%)	51.7 ± 29.1	36.8 ± 27.7	25.6 ± 24.7
*AUC*_PT_ (%*h)	212 ± 128	158 ± 150	132 ± 158

**Table 3:** Summary of model building steps for the pharmacokinetics of RIV

Model Building Step	Model No.	Description		Df	OFV	ΔOFV	Compared to Model No.
Step 1. Development of a base model. Dataset: PKs of RIV administered alone (GR1)	1.1	1-compartment model	First-order kinetics	-	860.38	-	-
	1.2		Zero-order kinetics	0	861.42	1.034	1.1
	1.3		Combined first- and zero-order kinetics	4	859.71	−0.669	1.1
	1.4	2-compartment model	First-order kinetics	-	858.71	-	-
	1.5		Zero-order kinetics	0	859.92	1.783	1.4
	1.6 ^¥^		Combined first- and zero-order kinetics	4	839.38	−18.75	1.4
	1.6 ^¥^		Combined first- and zero-order kinetics	1	839.38	−21.00	1.1
Step 2. Development of covariate models. Dataset: Full PKs of RIV (GR1, GR2, GR3)	1.6 ^¥^	Base model		-	1470.68	-	-
	2.1	Base model with addition of IIV for *D2*		1	1460.11	−10.56	1.6
	2.2	Model 2.1 with addition of a covariate for *D2*		2	1435.24	−24.88	1.6
	2.3	Model 2.1 addition of a covariate for *CL/F*		2	1419.07	−41.05	1.6
	2.4	Model 2.1 with addition of a covariate for both *CL/F* and *D2*		2	1397.64	−21.42	2.3
	2.4	Final covariate model		4	1397.4	−73.04	1.6

^¥^ final base model; OFV, objective function value; ΔOFV, change in objective function value; Df, degree of freedom. GR1, RIV alone; GR2, RIV administered with CBZ; GR3, RIV administered with PHT.

**Table 4:** Parameter estimates from the final covariate model and results of bootstrap validation for RIV.

Parameters	Unit	Estimates	RSE (%)	Shrinkage (%)	Bootstrap Replicates (*n* = 1000)			
					Median	95% CI		
*TVCL/F*	L/h/kg	0.61	36.9		0.609	0.456	−	0.817
$FCL_{CBZ}$		2.11	69.7		2.11	1.15	−	3.48
$FCL_{PHT}$		10.30	71.8		10.9	6.28	−	17.4
*TV* $D_{2}$	h	6.62 (FIXED)	N.E.		6.62	6.62	−	6.62
$FD2_{CBZ}$		0.339	17.2		0.335	0.255	−	0.380
$FD2_{PHT}$		0.434 (FIXED)	N.E.		0.434	0.434	−	0.434
$V_{c}$ */F*	L/kg	0.693	44.2		0.701	0.514	−	1.06
*Ka*	1/h	2.27	33.6		2.31	1.46	−	3.11
*Q/F*	L/h/kg	0.660	21.4		0.665	0.520	−	0.875
$V_{p}$ */F*	L/kg	5.57	38.1		5.60	3.83	−	8.93
*F1*		0.250	9.10		0.260	0.211	−	0.351
*Alag2*	h	0.501 (FIXED)	N.E.		0.501	0.501	−	0.501
Inter-individual variability								
IIV for $V_{c}$*/F*	%	47.0	33.0	4.0	44.5	30.9	−	59.7
IIV for *CL/F*	%	49.0	35.0	23.0	46.7	19.2	−	63.3
Residual random variability								
Additive error	ng/mL	13.6	35.8		13.5	6.33	−	23.7
Proportional error	%	23.2	17.2		22.0	16.0	−	26.1

RSE, relative standard error; IIV, inter-individual variation; CI, confidence interval; *TVCL*, the typical clearance value of RIV in population; N.E., not estimated; $FCL_{CBZ}$ and $FCL_{CBZ}$, the fractional change in the clearance of RIV in subjects coadministered with CBZ (GR2) or PHT (GR3); *TVD2*, the typical duration absorption time of RIV (describing the absorption rate of RIV following the zero-order kinetics) in population; $FD2_{CBZ}$ and $FD2_{CBZ}$, the fractional change in the duration absorption time of RIV in subjects coadministered with CBZ (GR2) or PHT (GR3); *Ka*, the absorption rate constant describing the absorption of RIV following the first-order kinetics; *F1*, the fraction of RIV absorbed following the first-order kinetics; $V_{c}$ and $V_{p}$, volume of distribution of RIV in the central and peripheral compartment, respectively; *Q*, inter-compartmental clearance; *Alag2*, the delay time from when the drug was administered until the absorption following the zero-order kinetics was started.

## Supplementary Materials

The following are available online at https://www.mdpi.com/1999-4923/12/11/1040/s1, Table S1: Parameter estimates from the final base model for RIV.
