# Supplementary material for: Effects of Carbamazepine and Phenytoin on Pharmacokinetics and Pharmacodynamics of Rivaroxaban
Source: Pharmaceutics. 2020 Oct 30;12(11):1040. doi: 10.3390/pharmaceutics12111040 (PMC7693231; doi:10.3390/pharmaceutics12111040)
Supplement: Supplementary file 1 [file pharmaceutics-12-01040-s001.pdf]

pharmaceutics

MDPI

Supplemaenatry Material

Effects of Carbamazepine and Phenytoin on Pharmacokinetics and Pharmacodynamics of Rivaroxaban

Lien Thi Ngo, Sung-yoon Yang, Quyen Thi Tran, Sang Kyum Kim, Hwi-yeol Yun and Jung-woo Chae

Table S1. Parameter estimates from the final base model for RIV.

Parameters	Description	Unit	Estimates	RSE (%)	Shrinkage (%)
D_2	Duration absorption time of the zero-order absorption	h	6.52	8.70	
Ka	Absorption rate constant of the first-order absorption	1/h	2.26	50.0	
CL/F	Apparent clearance	L/h/kg	1.58	74.7	
V_c/F	Apparent volume of distribution in the central compartment	L/kg	0.965	69.4	
Q/F	Apparent intercompartment clearance	L/h/kg	0.773	44.5	
V_p/F	Apparent volume of distribution in the peripheral compartment	L/kg	6.43	93.9	
F1	The fraction of RIV absorbed following the first-order kinetics		0.32	21.4	
Alag2	The delay time of the zero-order absorption	h	0.55	75.8	
Interindividual variability (IIV)					
IIV for V_c/F		%	44.0	31.2	21.8
IIV for *CL/F*		%	129.6	61.6	1.4
Residual random variability					
Additive error		ng/mL	31.2	11.2	
Proportional error		%	17.7	23.3	
